# Treatment Patterns and Use of Resources in Patients With Tuberous Sclerosis Complex: Insights From the TOSCA Registry

**DOI:** 10.3389/fneur.2019.01144

**Published:** 2019-10-25

**Authors:** Ruben Marques, Elena Belousova, Mirjana P. Benedik, Tom Carter, Vincent Cottin, Paolo Curatolo, Maria Dahlin, Lisa D'Amato, Guillaume Beaure d'Augères, Petrus J. de Vries, José C. Ferreira, Martha Feucht, Carla Fladrowski, Christoph Hertzberg, Sergiusz Jozwiak, John A. Lawson, Alfons Macaya, Rima Nabbout, Finbar O'Callaghan, Jiong Qin, Valentin Sander, Matthias Sauter, Seema Shah, Yukitoshi Takahashi, Renaud Touraine, Sotiris Youroukos, Bernard Zonnenberg, John C. Kingswood, Anna C. Jansen

**Affiliations:** ^1^Novartis Farma SpA, Origgio, Italy; ^2^Institute of Biomedicine (IBIOMED), University of Leon, León, Spain; ^3^Research and Clinical Institute of Pediatrics, Pirogov Russian National Research Medical University, Moscow, Russia; ^4^SPS Pediatrična Klinika, Ljubljana, Slovenia; ^5^TSA Tuberous Sclerosis Association, Nottingham, United Kingdom; ^6^Hôpital Louis Pradel, Claude Bernard University Lyon 1, Lyon, France; ^7^Tor Vergata University Hospital, Rome, Italy; ^8^Karolinska University Hospital, Stockholm, Sweden; ^9^Association Sclérose Tubéreuse de Bourneville, Gradignan, France; ^10^Division of Child and Adolescent Psychiatry, University of Cape Town, Cape Town, South Africa; ^11^Centro Hospitalar Lisboa Ocidental, Lisbon, Portugal; ^12^Medical University of Vienna, Universitätsklinik für Kinder-und Jugendheilkunde, Vienna, Austria; ^13^Associazione Sclerosi Tuberosa ONLUS, Milan, Italy; ^14^European Tuberous Sclerosis Complex Association, In den Birken, Dattein, Germany; ^15^Vivantes-Klinikum Neukölln, Berlin, Germany; ^16^Department of Child Neurology, Warsaw Medical University, Warsaw, Poland; ^17^Department of Neurology and Epileptology, The Children's Memorial Health Institute, Warsaw, Poland; ^18^The Tuberous Sclerosis Multidisciplinary Management Clinic, Sydney Children's Hospital, Randwick, NSW, Australia; ^19^Hospital Universitari Vall d'Hebron, Barcelona, Spain; ^20^Department of Pediatric Neurology, Necker Enfants Malades Hospital, Imagine institute Inserm 1163, Paris Descartes University, Paris, France; ^21^Institute of Child Health, University College London, London, United Kingdom; ^22^Department of Pediatrics, Peking University People's Hospital (PKUPH), Beijing, China; ^23^Tallinn Children Hospital, Tallinn, Estonia; ^24^Klinikverbund Kempten-Oberallgäu gGmbH, Kempten, Germany; ^25^Novartis Healthcare Pvt. Ltd., Hyderabad, India; ^26^National Epilepsy Center, Shizuoka Institute of Epilepsy and Neurological Disorders, NHO, Shizuoka, Japan; ^27^Department of Genetics, CHU-Hôpital Nord, Saint Etienne, France; ^28^St. Sophia Children's Hospital, Athens, Greece; ^29^University Medical Center, Utrecht, Netherlands; ^30^Cardiology Clinical Academic Group, Molecular and Clinical Sciences Research Centre, St Georges University of London, London, United Kingdom; ^31^Pediatric Neurology Unit, Department of Pediatrics, Universitair Ziekenhuis Brussel, Vrije Universiteit Brussel, Brussels, Belgium

**Keywords:** TSC, resource use, TOSCA, management, registry, rare diseases

## Abstract

Tuberous Sclerosis Complex (TSC) is a rare autosomal-dominant disorder caused by mutations in the *TSC1* or *TSC2* genes. Patients with TSC may suffer from a wide range of clinical manifestations; however, the burden of TSC and its impact on healthcare resources needed for its management remain unknown. Besides, the use of resources might vary across countries depending on the country-specific clinical practice. The aim of this paper is to describe the use of TSC-related resources and treatment patterns within the TOSCA registry. A total of 2,214 patients with TSC from 31 countries were enrolled and had a follow-up of up to 5 years. A search was conducted to identify the variables containing both medical and non-medical resource use information within TOSCA. This search was performed both at the level of the core project as well as at the level of the research projects on epilepsy, subependymal giant cell astrocytoma (SEGA), lymphangioleiomyomatosis (LAM), and renal angiomyolipoma (rAML) taking into account the timepoints of the study, age groups, and countries. Data from the quality of life (QoL) research project were analyzed by type of visit and age at enrollment. Treatments varied greatly depending on the clinical manifestation, timepoint in the study, and age groups. GAB Aergics were the most prescribed drugs for epilepsy, and mTOR inhibitors are dramatically replacing surgery in patients with SEGA, despite current recommendations proposing both treatment options. mTOR inhibitors are also becoming common treatments in rAML and LAM patients. Forty-two out of the 143 patients (29.4%) who participated in the QoL research project reported inpatient stays over the last year. Data from non-medical resource use showed the critical impact of TSC on job status and capacity. Disability allowances were more common in children than adults (51.1% vs 38.2%). Psychological counseling, social services and social worker services were needed by <15% of the patients, regardless of age. The long-term nature, together with the variability in its clinical manifestations, makes TSC a complex and resource-demanding disease. The present study shows a comprehensive picture of the resource use implications of TSC.

## Introduction

Tuberoussclerosis complex (TSC) is an autosomal-dominant disorder characterized by the formation of hamartomatous lesions in multiple organ systems (1) and the association with a wide range of TSC-associated neuropsychiatric disorders, abbreviated as TAND (2).

TSC is caused by mutations in either *TSC1* or *TSC2* genes. The proteins encoded by these two genes—hamartin and tuberin—form a complex that inhibits the mammalian target of rapamycin (mTOR) complex 1, which is involved in the regulation of cell growth and proliferation (1).

The manifestations and the severity of the disease are variable, even between relatives, and depend on size, number, location and distribution of the lesions (3, 4). Common locations include the brain, kidneys, lungs, skin, heart, and eyes (4–8). However, no single symptom is observed in all patients, and none of the symptoms can be considered as absolutely pathognomonic (6).

The use of resources and the costs of managing patients with TSC have been estimated in several studies carried out in Sweden (9), the United Kingdom (UK) (10–12), the Netherlands (13), the United States (US) (14, 15), and Canada (16). All of them have been developed on a national-basis in European countries or in North America, and most of them have been carried out in a limited number of patients filtered by age or by clinical manifestation. Therefore, the information coming from these studies is specific and cannot be completely extrapolated to other countries or clinical contexts. High variations across countries can appear depending on the country-specific clinical practice. As a consequence, the burden of TSC and its impact on the use of healthcare resources required for its management remain unknown.

The TuberOus SClerosis registry to increase disease Awareness (TOSCA) was a large scale non-interventional study in patients with TSC, started in 2012 and was conducted at 170 sites in 31 countries. TOSCA registry was totally founded by Novartis AG and its related clinical study protocol and final study results are disclosed on the ENCePP portal at http://www.encepp.eu/ (EU PAS Register Number EUPAS324) (17). The design and methodology of TOSCA were published previously (8). In short, patients of any age with TSC were enrolled and followed-up for up to 5 years. Patient data including demographics and information related to clinical features of TSC across all organ systems, comorbidities, and rare manifestations, were collected at baseline and at regular visits scheduled at a maximum interval of 1 year.

The registry consisted of a “core” part and six associated research projects focusing on: epilepsy, subependymal giant cell astrocytomas (SEGA), renal angiomyolipoma (rAML)/lymphangioleiomyomatosis (LAM), genetics, quality of life (QoL), and TSC-associated neuropsychiatric disorders (TAND); the “core” part collected demographic data, family history, prenatal history, disease features, and information on treatments, whereas the research projects recorded in-depth data related to specific disease manifestations or to specific aspects of the disease (8). One of the research projects (research project on QoL) recorded data on the use of medical and non-medical resources for seven European countries (Belgium, Germany, Italy, Spain, Sweden, France, and the UK).

Due to its long-term follow-up (up to 5 years) and to the inclusion of patients of any age from different countries from all over the world, the TOSCA registry offered a unique opportunity to observe how treatment patterns for the manifestations of TSC changed over time, and to evaluate differences in disease management depending on the age of the patients or their country of residence. In addition, results can be analyzed in context with the results from the other research projects.

The aims of the present study were to analyse how the treatment modalities in patients with TSC included in the TOSCA registry changed during the 5 years of follow-up, to identify differences in management as well as the availability of medical and non-medical health resources with respect to patients' age or country of residence.

## Methods

This study was based on data obtained from the TOSCA registry. The TOSCA registry was a non-interventional clinical study founded by Novartis AG, designed and conducted according to the Guidelines for Good Clinical Practice and ethical principles outlined in the Declaration of Helsinki (18, 19). After appropriate approval by central and local research ethics committees, written informed consent was obtained from all patients, parents, or guardians, prior to enrollment.

The first step for the present manuscript was a search for variables that could be of interest for the purpose of a study on the use of TSC-related resources (including medical and non-medical resources), and an exhaustive analysis of all the listings and tables produced as part of the final analysis of the TOSCA registry, in order to identify relevant outcomes and analyses for each variable. The variables and potential analyses are detailed in the Tables S1, S2.

Data on use of treatments (proportion of treated patients and types of treatment) were available for the overall population of patients included in the core registry. Data on the use of other medical resources (hospitalizations, primary, and secondary care visits) and on the use of non-medical resources (variables related to education needs, patient or caregiver employment situation and patient support/social services needs) were available for a subset of 143 patients included in the QoL research project, which was carried out in 7 European countries (Belgium, Germany, Italy, Spain, Sweden, France, and the UK).

Treatment patterns were analyzed using the core registry data according to 4 clinical manifestations (epilepsy, SEGA, LAM, and rAML), the number of visits [baseline or follow-ups (FU1 to FU5), where FUs were conducted at intervals not longer than 12 months apart], the age group (≤2, >2 to ≤ 5, >5 to ≤9, >9 to ≤14, >14 to <18, ≥18 to ≤40, and >40 years), and the country of residence (for those countries included in the QoL research project; i.e., Belgium, Germany, Italy, Spain, Sweden, France, and the UK). Baseline data were retrospectively collected and FU data were prospectively collected up to 5 years. All the results were reported in terms of absolute and relative frequencies.

The use of other medical resources and the use of non-medical resources was analyzed for the overall population included in the QoL research project. Again, all the results were reported in terms of absolute and relative frequencies.

## Results

### Baseline Characteristics of Patients

The baseline characteristics of patients enrolled in TOSCA registry were analyzed in detail. In brief, a total of 2,214 patients from 31 countries worldwide were enrolled into the study. Data from 2,211 eligible patients were analyzed as part of the TOSCA clinical study report delivered to Health Authorities by Novartis AG. Data of 3 patients were excluded from the analysis because of major protocol deviations. Of the analyzed patients, 1,152 (52.1%) were female. The median age at enrolment was 13 years (range <1–71), and the median age at first TSC diagnosis was 1 year (range <1–69 years). The most common manifestation was epilepsy occurring in 1,879 (85.0%) of patients. Among patients with epilepsy, 1,343 (71.5%) had focal seizures (FS) and 735 (39.1%) had infantile spasms (IS). Other common manifestations were hypomelanotic macules in 1,555 patients (70.3%), facial angiofribromas in 1,533 patients (69.3%), and rAML in 1,317 patients (59.6%).

Another important manifestation was TAND, even though it was the most underassessed aspect of TSC in the registry. TAND assessment includes the evaluation of common behavioral problems, psychiatric disorders, intellectual abilities, academic performance, and neuropsychological difficulties. At baseline, only 818 out of 2,211 (37%) patients reported to have at least one behavioral problem, in 319 (14.4%) patients autism spectrum disorder (ASD) and in 267 (12.1%) patients attention deficit hyperactivity disorders (ADHD) was diagnosed, and 82 (3.7%) and 132 (6.0%) patients had depressive disorders or anxiety, respectively. In addition, 736 patients (33.3%) were reported to have difficulties in academic performance. Among the 894 patients with reported TAND, normal intellectual ability (defined as full scale IQ ≥80) was reported for 44.2% (395/894).

### Treatments

**Figure 1:**
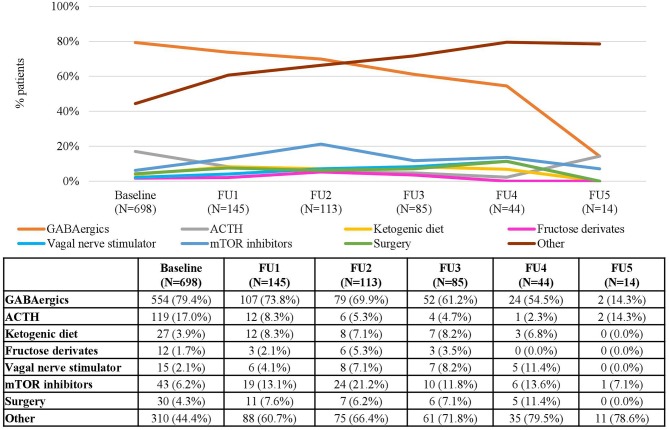
Treatments for Infantile Spasms in each Follow-up Visit. Patients may receive more than one treatment. Baseline data refers to patients who “ever had” the manifestation. Other include lamotrigine, topiramate, levetiracetam and valproate.

**Figure 2:**
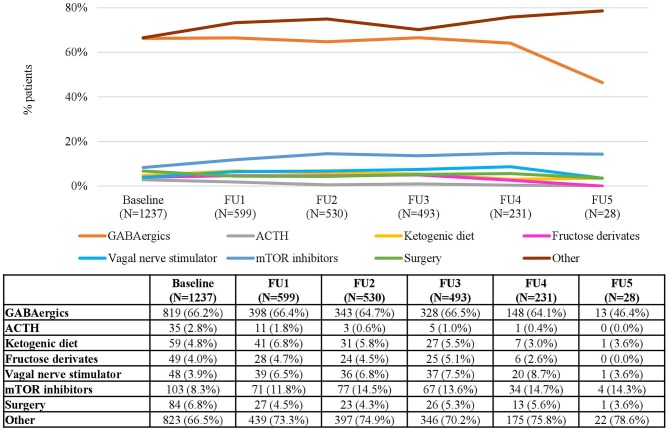
Treatments for Focal Seizures in each Follow-up Visit. Patients may receive more than one treatment. Baseline data refers to patients who “ever had” the manifestation.

In the TOSCA registry, the proportion of patients who received treatment varied largely depending on the clinical manifestations (Table 1), with values at baseline (patients who ever had the manifestation) ranging between 96.8% (698/721) for IS and 32.5% (50/154) for LAM. Almost all patients with epilepsy received antiepileptic drug treatment without relevant variations throughout the study (Table 1). At baseline, the most common treatments were GABAergic agents (e.g., vigabatrin), both in mono- and combination therapy), which were used in 79.3% of treated patients with IS, and in 66.2% of treated patients with FS (Figures 1, 2).

**Table 1:** Use of treatments according to follow-up visit.

	**Baseline (*N* = 2211)**	**FU1 (*N* = 2099)**	**FU2 (*N* = 1935)**	**FU3(*N* = 1664)**	**FU4 (*N* = 764)**	**FU5 (*N* = 147)**
Patients with IS	721	151	120	91	45	14
Patients treated for IS (*n*, %)	698 (96.8)	145 (96.0)	113 (94.2)	85 (93.4)	44 (97.8)	14 (100.0)
Patients with FS	1,261	614	544	506	236	29
Patients treated for FS (*n*, %)	1,237 (98.1)	599 (97.6)	530 (97.4)	493 (97.4)	231 (97.9)	28 (96.6)
Patients with SEGA	553	489	468	420	208	52
Patients treated for SEGA (*n*, %)	221 (40.0)	187 (38.2)	188 (40.2)	181 (43.1)	101 (48.6)	22 (42.3)
Patients with rAML	1,062	1,067	1,041	945	472	121
Patients treated for rAML (*n*, %)	315 (29.7)	300 (28.1)	321 (30.8)	288 (30.5)	165 (35.0)	53 (43.8)
Patients with LAM	154	157	162	149	68	21
Patients treated for LAM (*n*, %)	50 (32.5)	47 (29.9)	54 (33.3)	43 (28.9)	20 (29.4)	0 (0.0)

However, the use of GABAergic agents decreased over time, reaching a minimum of 14.3% in the fifth FU visit for the IS patients and 46.4% for FS patients. Other treatment options such as mammalian target of rapamycin (mTOR) inhibitors, the ketogenic diet (KD) and epilepsy surgery were used in <20% of the patients at baseline, and remained relatively stable over time (Figures 1, 2).

**Figure 3:**
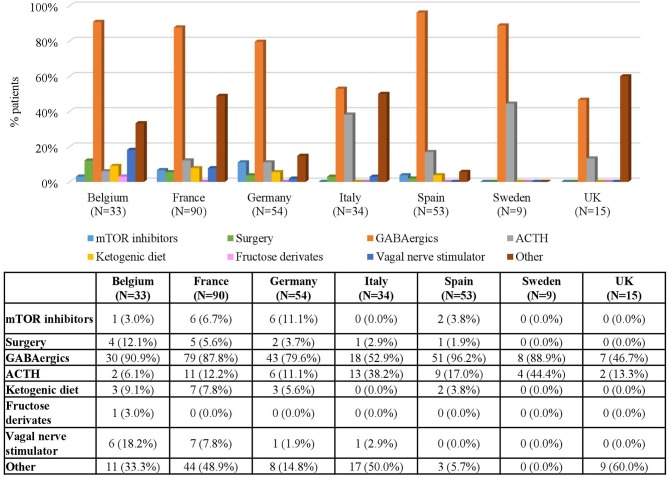
Treatments for Infantile Spasms by Country. Patients may receive more than one treatment.

**Figure 4:**
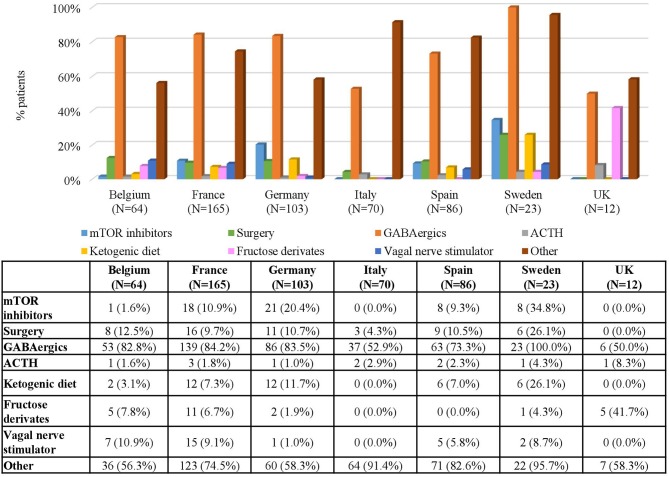
Treatments for Focal Seizures by Country. Patients may receive more than one treatment.

When analyzing the types of treatment by country, GABAergics alone or in combination were by far the most common treatment options in all countries both in patients with IS (ranging between 46.7% in the UK and 96.2% in Spain) and in patients with FS (ranging between 50% in the UK and 100% in Sweden) (Figures 3, 4). Adrenocorticotropic Hormone (ACTH) was the second most common treatment for treating IS in all countries except in Belgium. Other common treatments for treating FS were epilepsy surgery (in Belgium, Italy, and Spain) and mTOR inhibitors (in Sweden, Germany, and France) (Figure 4). Of note, both surgery and mTOR inhibitors were not used at all in patients with IS or FS from the UK, and in patients with IS from Sweden. More than 50% of the treatments in patients with FS were not specified (included in “others” category) in all countries, even more than 90% in Italy and Sweden (Figure 4).

**Figure 5:**
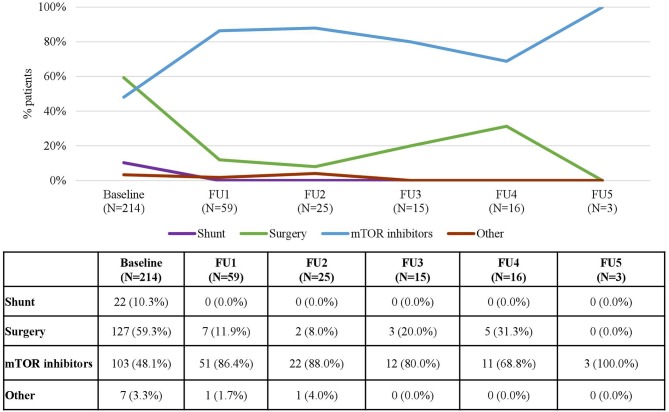
Treatments for SEGA in each Follow-up Visit. Patients may receive more than one treatment. Baseline data refers to patients who “ever had” the manifestation.

At baseline, 40.0% of patients had ever received treatment for SEGA and this proportion remained stable over time (Table 1). mTOR inhibitors and surgery were the most common procedures in patients with SEGA with marked differences depending on follow-up, age and the country of residence (Figure 5). At baseline, mTOR inhibitors were administered in 48.1% of the patients who received treatment for SEGA, but their use increased over time (reaching 86.4% of patients in the 1st FU visit and 100% in the 5th). In contrast, 59.3% patients received surgery at baseline, but the proportion of patients undergoing surgery decreased over time as the use of mTOR inhibitors increased (reaching 11.9% of patients in the 1st FU visit and no patients in the 5th) (Figure 5).

**Figure 6:**
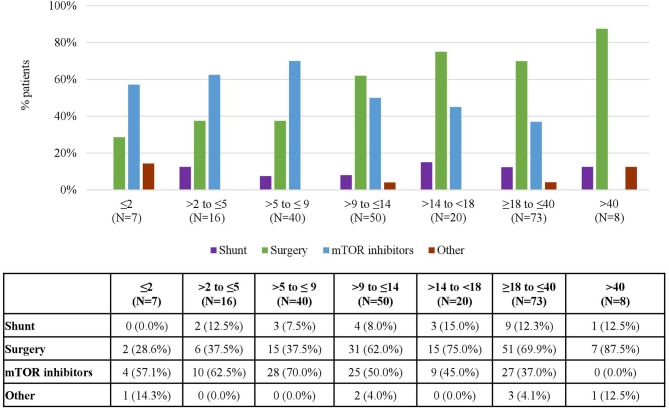
Treatments for SEGA according to Age at Baseline. Patients may receive more than one treatment.

The proportion of patients treated for SEGA also varied depending on the age at baseline. Children aged 9–14 were treated most commonly [50 (51.0%) patients received treatment] while children aged <2 years and adults aged more than 40 years were treated least frequently [7 (15.2%) and 8 (29.6%) of patients, respectively]. Likewise, the types of treatment varied across age groups. While mTOR inhibitors were the most common treatments used in children aged 9 or less [reaching a peak (70%) in those aged between 5 and 9], surgery was the most common treatment in adolescents and adults [reaching a peak (87.5%) in those aged more than 40] (Figure 6).

**Figure 7:**
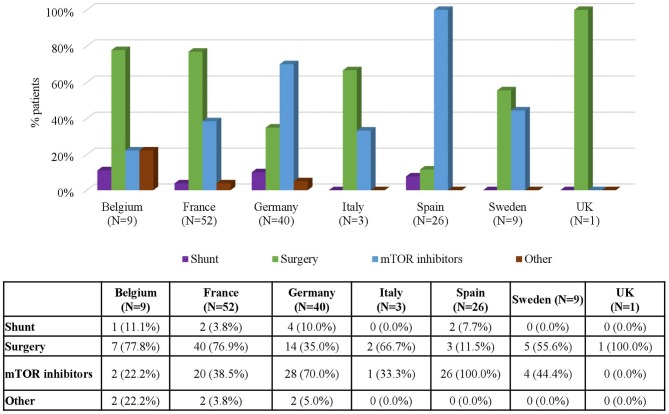
Treatments for SEGA by Country. Patients may receive more than one treatment.

Regarding the use of treatments for SEGA by country, mTOR inhibitors were more often prescribed in Germany (70% of the patients) and Spain (100% of the patients) than in the rest of the participating countries (Figure 7). In contrast, surgery was the most common treatment in Belgium (77.8%) and in France (76.9%). The only patient from the UK (100%) also underwent surgery.

**Figure 8:**
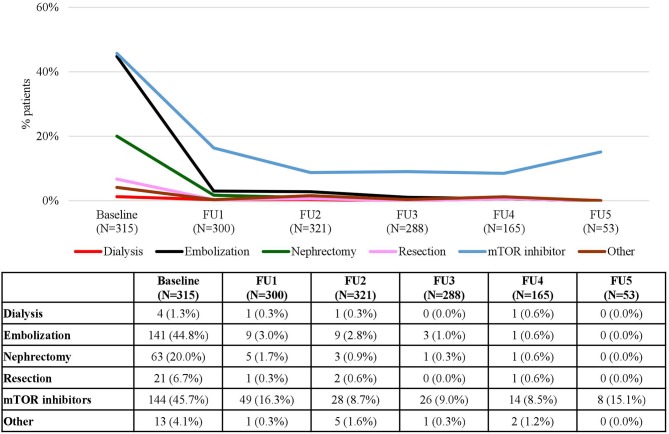
rAML Treatments according to Follow-Up. Patients may receive more than one treatment. Baseline data refers to patients who “ever had” the manifestation.

**Figure 9:**
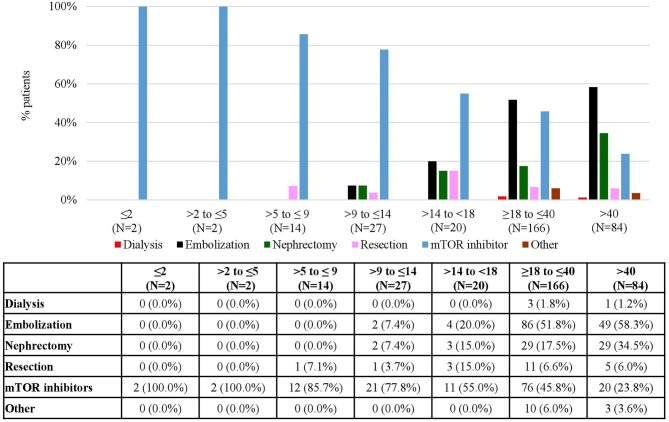
rAML Treatments according to Age at Baseline. Patients may receive more than one treatment.

**Figure 10:**
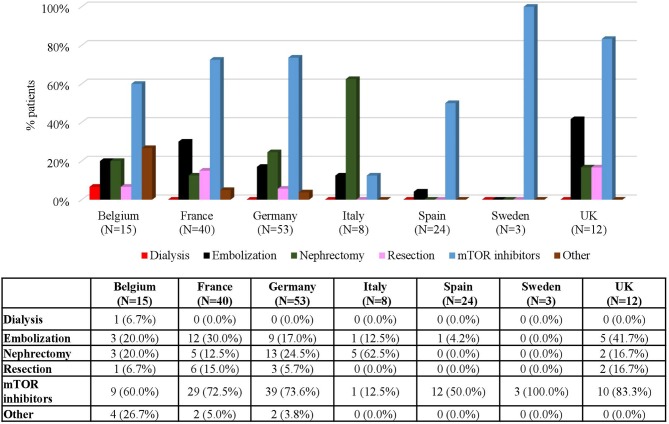
rAML Treatment by Country. Patients may receive more than one treatment.

With respect to rAML, the number of patients treated was 315 (29.7%) at baseline, kept at around 30% up to FU 3 and increased in FU 4 (35.0%) and FU 5 (43.8%) (Table 1). Similarly to SEGA, mTOR inhibitors and embolization were the most common treatments for rAML patients (Figure 8). At baseline, 144 (45.7%) patients received mTOR inhibitors and 141 (44.8%) patients underwent embolization; however, the use of all treatments consistently decreased with time with only 8 (15.1%) patients in FU 5 receiving mTOR inhibitors. Data on embolizations were not available for any patient at the end of the period and only one patient (0.6%) underwent this procedure in FU 4 (Figure 8). rAML is an uncommon manifestation in children. Therefore, most of the patients receiving treatment for rAML were adolescent and adults (Figure 9). Embolizations were rare in children (only 7.4% of patients aged 9–14 had undergone this procedure) whereas more than half of rAML patients aged 18–40 (51.8%) and older (58.3%) underwent this procedure. In contrast, there was a high use of mTOR inhibitors for rAML in these young patients, which certainly was prescribed for other TSC manifestations, which decreased for older patients (Figure 9). The distribution of treatments by country is shown in Figure 10. It can be observed that mTOR inhibitors were the most commonly used treatment option for rAML in all countries (Figure 10).

**Figure 11:**
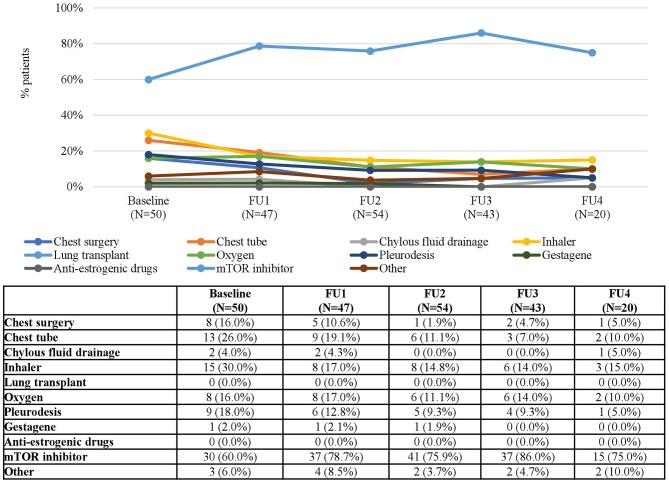
LAM Treatments according to Follow-Up. Patients may receive more than one treatment. Baseline data refers to patients who “ever had” the manifestation.

**Figure 12:**
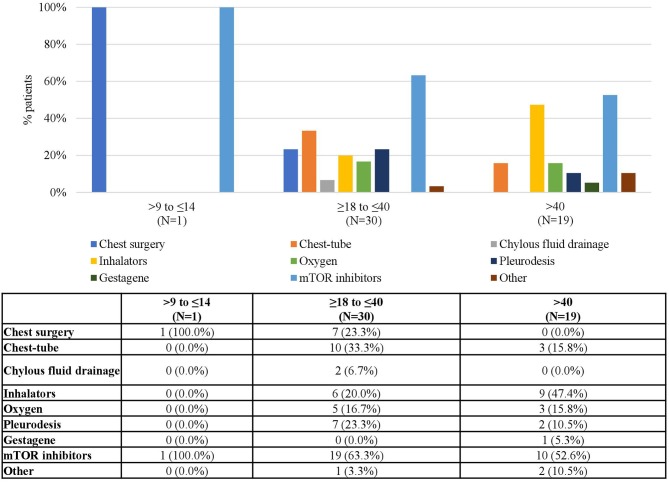
LAM Treatments according to Age at Baseline. Patients may receive more than one treatment.

As for LAM, the number of treated patients generally decreased with time (Table 1). Again, mTOR inhibitors were the most common treatment for this condition (60.0% of LAM patients received mTOR inhibitors at baseline) and its use increased up to 86.0% in FU 3 and 75.0% in FU4 (Figure 11). Since, as expected, LAM was only diagnosed in patients aged ≥9 years, no data were available for younger patients. Adolescents were treated with both chest surgery and mTOR inhibitors, while most patients treated during adulthood received mTOR inhibitors (Figure 12).

**Figure 13:**
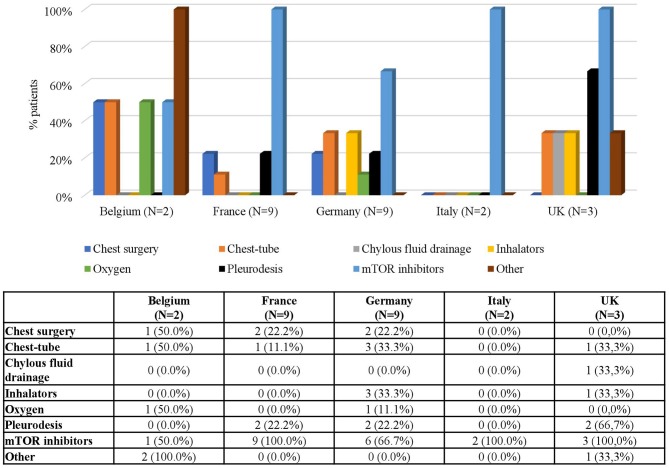
LAM Treatments by Country. Patients may receive more than one treatment.

mTOR inhibitors were used for LAM treatment in all patients in France and in Italy, in 66.7% in Germany, 50% in Belgium, and in 25.0% in the UK. No data on the type of treatments used in patients with LAM were available for Spain and Sweden (Figure 13).

### Hospitalizations and Visits

The frequency of hospitalizations was analyzed in the subset of patients of the TOSCA registry included in the QoL research project (*N* = 143). Regarding visits to the specialist, the same subset was analyzed. Subjects from Spain (*N* = 11) were excluded from the analysis because of data inconsistencies in these patients. As a result, healthcare visits were analyzed in 132 patients. A total of 88 visits to the specialists were reported over 12 months during the last year. Half of the patients (69/132; 52.3%) visited the specialist due to TSC at least once during the last year, and a quarter (29/132; 22.0%) had 3 or more visits. Visits to the specialist for reasons other than TSC were reported for 34 patients (25.8%), and 14 of them (10.6%) reported 3 or more visits during the last year (Table S3). Visits to the general practitioner (GP) were discarded from the analysis because of missing data (information was missing or unknown for more than 50% of the patients).

No hospitalizations were reported for 70.6% of the patients over 12 months during the last year. A third of the patients (41/143; 28.7%) reported at least one hospitalization, and 6.3% (9/143) reported 3 or more hospitalizations (Table S4).

Information on the use of non-medical resources (education, employment, use of social services and patient support requirements) was collected within the QoL research project, and this is summarized in Table S5.

Regarding education, 28 children (31.8%) were not in a mainstream school, and the rest (*N* = 57; 64.8%) were educated in a mainstream school. Of those who attended a mainstream school, 64.9% received special education within the school, and for 45.6% (26/57) the school offered special programs adequate to their condition (Table S5).

In the questionnaire used for data collection into this research project, 55 adults with TSC who were able to complete the questionnaire themselves and 88 carers for children with TSC reported their work experience. Only half of the individuals [41.8% (23/55) adult patients and 65.9% (58/88) children's carers] reported to have a job. A quarter of the adult patients (14/55; 25.5%) reported that they were not able to work due to TSC and half (28/55; 50.9%) stated that TSC had an impact on their career. The corresponding figures for these two items in children's carers were 9.1% (8/88) and 56.8% (50/88) (Table S5).

Besides, half of the children (45/88; 51.1%) and 38.2% (21/55) of the adults received a disability allowance, and 20% (11/55) of the adults received support with daily activities. Other services such as psychological counseling, social services, and social worker services were received by <15% of the patients irrespective of their age (Table S5).

## Discussion

The present work investigated treatment patterns and use of medical/non-medical resources in patients enrolled into the TOSCA registry. Compared to other studies carried out in single countries including a limited number of patients of certain age-groups or with specific manifestations (9–16, 20), the TOSCA registry represented a unique opportunity to analyse the treatment patterns and use of resources in a large cohort of pediatric and adult patients with a wide range of clinical manifestations who had been diagnosed and treated in different countries over a 5-year observation period. This strengthens the external validity of the results and provides clues on how treatment patterns have changed over time and across regions.

One of the purposes of the 2012 International TSC Consensus Conference was to provide recommendations for standardized diagnostic criteria, management and surveillance of TSC regardless of age (21). This study shows that treatment patterns mostly depend on the clinical manifestations of the disease but also that they depend on the age and the country of residence of the patients. For instance, there are important variations in the use of mTOR inhibitors in patients with SEGA throughout countries (ranging from none in the UK to 100% in Spain), and on the age of the patients (ranging from 70% in patients aged 5–9 to 0% in patients aged >40).

The differences between countries reflect not only the effect of clinical practice, but also the effect of access barriers due to different time points at which mTOR inhibitors were available for the various indications in specific countries and/or healthcare systems. For instance, everolimus was reimbursed for patients with FS in January 2017 in Germany and April 2018 in Sweden, but was not made available until late 2018 or the beginning of 2019 in the rest of European countries (June 2018 in Spain, September 2018 in Italy, December 2018 in Belgium, and in the UK, and January 2019 in France). For patients with SEGA, everolimus was reimbursed in October 2011 in Germany and in the UK only through the Individual Funding Request (IFR) route, while it was not available until 2016 in Italy and in Belgium. Another example is the availability of mTOR inhibitors for patients with rAML as everolimus was reimbursed in the UK in October 2011, in Germany in November 2012, and in France in April 2014, even though it was not available in Spain until April 2015 and in Belgium until August 2016, and it is still not yet reimbursed in Italy.

In addition, the differences in age groups might reflect differences in clinical practice between pediatric and adult neurologists in those manifestations treated before the TOSCA registry and within the time horizon of the TOSCA registry (i.e., after baseline). In line with the current guidelines (21, 22), which recommend the use of vigabatrin as a first-line antiepileptic drug treatment in patients with TSC and either IS or FS before the age of 1 year, the most prescribed drugs were GABAergics. In any case, these results must be interpreted with caution due to the large proportion of treatments included in the category “others” (at baseline, 44.4% for IS and 66.5% for FS) and to the fact that the category “GABAergics” included a large number of different AEDs. In future studies, more attention should therefore be paid to the definition of treatment variables.

Besides, one has to take into consideration, that TOSCA enrollment started in August 2012, and last data entry was in August 2017. Everolimus, was approved by European Medicines Agency (EMA) for the treatment of drug-resistant epilepsy as late as in January 2017. It was therefore not possible to evaluate the consequences of the approval of this mTOR inhibitor on the treatment patterns of patients with TSC-associated epilepsies. Despite this, physicians struggling to treat TSC-associated seizures that had proved refractory to conventional AED treatment had already started using everolimus with increasing frequency. We hypothesize that this use was due to other TSC-associated conditions and on-going mTOR studies in epilepsy.

This study shows how mTOR inhibitors have become common treatments for a variety of manifestations in patients with TSC such as SEGA, LAM, and rAML. However, since more than one manifestation might co-occur in a single patient, it may not be correct to attribute the use of mTOR inhibitors to a single manifestation. An example of this is the use of mTOR inhibitors in patients with LAM as a consequence of the growing use of mTOR inhibitors for other indications in patients with TSC.

In patients with SEGA, current recommendations propose the use of surgical resection for acutely symptomatic SEGA, the use of both surgery and mTOR inhibitors for growing but asymptomatic SEGA and the use of mTOR inhibitors for patients with large or bilateral SEGA that are not amenable to surgical resection (21, 23). In line with the recommendations, the analyses on the use of treatment according to FU visits, countries, and age groups in the patients included in the TOSCA registry show that the increases in the use of mTOR are often accompanied by decreases in the use of surgery. For instance, it is particularly striking to observe how the increasing use of mTOR inhibitors registered in the different FU visits (Figure 5) is almost a mirror image of the decreasing use of surgery, and to observe how in age groups and countries where mTOR inhibitors are used the most, surgery is used the least and vice versa (Figures 6, 7).

The exact economic cost of these changes was not possible to evaluate from this dataset. However, the potential reductions and delays in the use of surgery may have economic implications not only at the time of treatment initiation, but also in the follow-up of the patients. In this regard, a study comparing pre-surgery and post-surgery costs in TSC patients with SEGA surgery carried out in the US (24) found that medication and total costs in the post-surgery year were 1.6–4.3 times the costs in the pre-surgery year. Unfortunately, no formal economic evaluations comparing surgery and mTOR inhibitors in patients with SEGA have been carried out.

Interestingly, the use of surgery in patients with SEGA was lower in the TOSCA registry (Figure 5) than in a previous survey study carried out by Rentz et al. (15). This study included 676 patients -or caregivers- and reported surgery in 31 and 47% of pediatric and adult patients, respectively, but did not report any use of mTOR inhibitors in any of the groups. Comparing the use of medical resources, and in particular the use of surgery depending on whether the patients receive treatment with mTOR inhibitors is an area of major interest that remains largely unexplored.

The results observed in rAML and LAM are in line with those observed in patients with SEGA. However, as stated above, since we are considering a population with co-occurring manifestations it is difficult to determine if mTOR inhibitors were used to treat these particular manifestations. It is worth commenting that in Sweden, where 100% of patients with rAML who received treatment received mTOR inhibitors, no patients had nephrectomy surgery; by contrast, in Italy, where only 12.5% of the patients who received treatment for this manifestation were treated with mTOR inhibitors, 62.5% had nephrectomy surgery (Figure 10).

While these results might also be influenced by the age of the patients in each country at baseline, it is important to emphasize that embolization surgery in rAML and chest surgery in LAM are rescue therapies in urgent situations, but mTOR inhibitors are the only available treatment that both modifies the disease and improves the outcomes (21, 25, 26).

A reason for the increased use of mTOR inhibitors in patients with LAM might be its inclusion in the recent international guidelines published for the diagnosis and management of LAM, in which mTOR inhibitors were recommended for patients with abnormal or declining lung function or with problematic chylous effusions, that could have affected the treatment patterns (27).

Given that TSC is a multi-organ disease, treatment of a certain manifestation with a systemic mTOR inhibitor will probably result in reductions of the use of surgical interventions for other manifestations as well. Concomitant systemic effects in patients treated with mTOR inhibitors have been reported (28). The impact of these effects on the use of other treatments or other medical resources have not yet been analyzed and is an interesting topic for future research. The consistent reductions in the use of surgery observed for all the manifestations in the present study support this hypothesis.

Similar to other studies (11, 15, 20), this study shows that patients with TSC are demanding healthcare resource users, but it also shows that the use of resources is not evenly distributed across patients and countries. In this regard, while a third of the patients included in the QoL research project did not attend any specialist due to TSC during the past year, a quarter of the patients had three or more visits in the same period. Likewise, while 71% of the patients were not hospitalized at any time, up to 6.3% were hospitalized three or more times during the past year. In future studies, it would be interesting to identify the clinical features of the patients who are likely to be more intense resource users in order to provide a better allocation of resources for the management of the disease.

The present study also shows that the impact of TSC on education and on employability is high. More than half of the children had special needs (were not in a mainstream school or received special education within their school), and unemployment rates were high both in patients and caregivers of children with TSC (34.1% in children's caregivers, and up to 50% in adults with TSC). Therefore, the economic impact of a TSC diagnosis is high for the patients and for their families. In line with these results, a multicenter French study that included adult patients with TSC and with a diagnosed epilepsy before 16 years old found that 52% of patients required special education programs and only 37% reported having a stable professional life, even though 65% of them had a salary below the minimum income threshold in France (29).

The rate of patients receiving psychological support was reportedly low both for adults and children. The same low rates were observed in the multicenter French study, where 35% of children and 13% of adults had a regular psychological follow-up (29). This contrasts with the expected rates of TAND and suggests that the psychological needs of patients are not being addressed properly. Of note, physicians' unawareness and no clear guidelines on TAND evaluation before 2013 might have led to more missing data, underestimating TAND difficulties. However, a set of consensus guidelines for the evaluation of neuropsychiatric problems had already been published in 2005 (30), suggesting that there was a lack of implementation of existing guidelines. Likewise, the proportion of patients receiving disability allowances was higher in children (51.1%) than adults (38.2%), the use of social worker services was reportedly lower in both children and adults (8.0% in children and 1.8 % in adults), and <10% of patients (5.7% of children and 3.6% of adults) reported to have received help while completing benefit applications. Altogether, these results indicate that many patients with TSC might be unaware of the possibility of receiving social services or that these services are not available in all the countries.

A strength of the TOSCA registry was the prospective follow-up of patients, which allowed to trace changes in treatment patterns over time. However, data from the two last follow-up visits (after 4 and 5 years) were available, for only 764 and 147 patients out of 2,211, respectively. Hence, caution is required when drawing conclusions from the last two visits. Although the number of patients in the last follow-up is relatively low compared to the patients for whom data was available at baseline, other studies on use of resources in patients with TSC have been carried out in patient cohorts with a smaller sample size. For instance, a study carried out by Skalicky et al. (20) included 116 patients and another study carried out by Lennert et al. (12) included only 95 patients.

The present study has some limitations. The main caveat was that data relating resource use from the QoL research project was collected for <10% of the patients included in the registry, which is in contrast with excellent data quality for the medical aspects of TSC recorded in the core study. This might be due to the fact that data collection of data into the QoL research study was not mandatory, due to the observative nature of the registry, or might be due to the absence of site monitoring review of the QoL research project data collection. Carrying out specific studies to broaden the evidence on the use of medical resources in patients with TSC remains an interesting topic for future research.

Also, the observational nature of the TOSCA registry meant that only available data from standard clinical practice was supposed to collected. As recruitment was made through centers with expertise in TSC, where mainly moderate-severe TSC manifestations are seen, milder cases could have been underestimated. Getting data from routine practice also meant discrepancies in some variables, as the way information is collected within centers is not homogeneous. In any case, the involvement of various centers and specialists has helped inclusion of a significant number of TSC patients, which should be representative of real clinical practice.

Unlike in other studies evaluating the costs of managing TSC manifestations carried out in a single country (10, 11, 13, 14, 16), costs estimations could not be performed given that the analyses were conducted using data from 31 countries with different healthcare systems.

Furthermore, there are differences between the design of this study and that of previous studies evaluating the use of resources in TSC patients (10–16, 20), which limits the conclusions that can be drawn when comparing our results. Besides the differences in geographical areas and timeframes, while the TOSCA registry included patients with proven TSC, but regardless of specific manifestations, only three of the studies published so far (11, 14, 15) were carried out in an overall TSC population (i.e., not defined by a specific manifestation), while the rest included only patients with epilepsy (10, 12), SEGA (20), LAM (16), or kidney involvement (13).

Our results show that the use of treatments for specific conditions greatly differed depending on the clinical manifestations and the specialists caring for the patients, the period analyzed, as well as their ages and the countries of residence. Therefore, comparing the results of the patients included in the TOSCA registry with those observed in other studies without paying attention to their baseline characteristics might be methodologically inappropriate.

Information about healthcare visits and hospitalizations, as well as about use of non-medical resources, was only available for a cohort of 143 patients from the 7 European countries included in the QoL research project. The fact that all the patients included in this project were treated in European countries limits the ability to extrapolate the conclusions to other continents. Also, some data inconsistencies were found regarding specialist visits in Spanish patients and the information regarding primary care (GP visits) was missing or unknown for half of the patients (50.3% for TSC-related visits and 53.9% for visits for other reasons). Future studies should incorporate monitoring strategies during data collection in order to minimize these issues.

Comparing the use of medical resources in patients with TSC treated with or without mTOR inhibitors remains another area of interest for future research. In addition, the information on medical and non-medical resources in the QoL research project was provided by the patient itself or a caregiver. Although this has been a common methodology in similar studies (10, 11, 15), there can be inconsistencies or missing data if patients do not remember the answers or do not understand the questions. Future research should pay attention to this point, involving specific staff to supervise data completion.

In conclusion, in spite of the limitations indicated above, this study has provided more detailed information about treatment patterns and current use of medical and non-medical resources in a large cohort of patients with TSC followed for a long period of time in seven European countries. It shows how mTOR inhibitors have become common treatments for certain TSC-related manifestations, often accompanied by reductions in the use of surgery. In addition, it confirms that the use of medical and non-medical resources in patients with TSC is high. Further research is needed to determine the impact of mTOR inhibitors on the use of other resources, and in particular, to quantify the economic consequences of potential reductions in the use of other treatments, primarily surgery.

## Data Availability Statement

Novartis supports the publication of scientifically rigorous analysis that is relevant to patient care, regardless of a positive or negative outcome. Qualified external researchers can request access to anonymized patient-level data, respecting patient informed consent, contacting study sponsor authors. The protocol can be accessed through EnCePP portal http://www.encepp.eu/ (EU PAS Register Number EUPAS3247).

## Ethics Statement

The study protocol and all amendments were reviewed and approved (if applicable) by independent ethics committee/institutional review board for each center: National Hospital Organization Central Ethics Committee, Gazi University Clinical Research Ethics Committee, Independent Multidisciplinary Committee on Ethical Review of Clinical Trials, Peking Union Medical College Hospital, Commissie Medische Ethiek UZ Brussel, CNIL (Commission National de l'Informatique et des Libertés), CCTIRS (Comité Consultatif sur le traitement de l'information en matière de recherche dans le domaine de la santé), Comité Etico Investigación Clínica de Euskadi (CEIC-E), Consejeria de Salud y Bienestar Social, Dirección General de Calidad, Investigación, Desarrollo e Innovación, Comité Coordinador de Ética de la Investigación Biomédica de Andalucía, Research Ethics Committee of the University of Tartu (UT REC), Ethikkommission der Medizinischen Universität Graz, North Wales REC – West, Regionala Etikprövningsnämnden i Göteborg, REK – Regionale komiteer for medisinsk og helsefaglig forskningsetikk, Komisja Bioetyczna przy Instytucie Pomnik Centrum Zdrowia Dziecka, Ethikkommission bei der Ludwig-Maximilians-Universitat München, Hokkaido University Hospital Independent clinical research Institutional Ethics Committee, Medical Juntendo University Institutional Ethics Committee, National Center for Chile Health and Deveropment of IRB, Osaka University Hospital of IRB, Ethics Committee at Moscow Institute of Pediatrics and Pediatric Surgery, Peking University First Hospital, Sanbo Brain Hospital Capital Medical University, Tianjin Children's Hospital, Childrens Hospital Of Fudan University, Zhongshan Hospital Fudan University, Fudan University Shanghai Cancer Center, The Second Affiliated Hospital of Guangzhou Medical University, The First Affiliated Hospital, Sun Yan-Sen University, The First Affiliated Hospital Of Guangzhou Medical University, Shenzhen Children's Hospital, West China Hospital, Sichuan University, Xijing Hospital, Children's Hospital of Chongqing Medical University, Wuhan Children's Hospital, The Second Affiliated Hospital of Xi'an Jiaotong University, Guangdong 999 Brain Hospital, Seoul National University Hospital Institutional Review Board, National Taiwan University Hospital (NTUH) Research Ethics Committee (REC), Institutional Review Board of the Taichung Veterans General Hospital, Institutional Review Board of Chung Shan Medical University Hospital, Institutional Review Board, Tungs' Taichung MetroHarbor Hospital, Institutional Review Board of National Cheng Kung University Hospital, Metro South Human Research Ethics Committee, Sydney Children's Hospital Network Human Research Ethics Committee, St Vincents Hospital Human Research Ethics Committee, Royal Melbourne Hospital Human Research Ethics Committee, Siriraj Institutional Review Board, The Institutional Review board, Faculty of Medicine, Chulalongkorn University, 3rd Floor, Ananthamahidol Building, King Chulalongkorn Memorial Hospital, The committee on Human Rights Related to Research Involving Human Subjects, Institutional Review board, Royal Thai Army Medical Department IRB RTA, 5th Floor, Phramongkutklaowejvitya Building, Phramongkutklao College of Medicine, Research Ethics Committee, Faculty of Medicine, Chiang Mai University, Research and Development, Queen Sirikit National Institute of Child Health, Human Research Ethics Committee, Faculty of Health Sciences, University of Cape Town, Shaare Zedek Meidcla Center Helsinki Comittee, Sheba Medical Center Helsinki Comittee, Tel Aviv Sourasly Medical Center Helsinki Comittee, General University Hospital of Patras Ethics Committee, Pendeli Children's Hospital Ethics Committee, General University Hospital of Athens G. Gennimatas Ethics Committee, Evaggelismos General Hospital Ethics Committee, General University Hospital of Thessaloniki AHEPA Ethics Committee, General University Hospital of Ionnina Ethics Committee, METC UMC Utrecht, Direcció General de Regulació, Planificació i Recursos Sanitaris, Comité Ético de Investigación Clínica del Hospital Universitario Vall d'Hebron de Barcelona, Generalitat de Catalunya. Departament de Salut, Comité Ético de Investigación Clínica Hospital Universitario La Paz, Dirección General de Ordenación e Inspección, Consejería de Sanidad Comunidad de Madrid, Servicios de Control Farmacéutico y Productos Sanitarios, Comité Etico Investigación Clínica del Hospital Universitario y Politécnico de La Fe, Dirección General de Farmàcia i Productes Sanitaris, Generalitat de Valencia, Comité de Ética de la Investigación de Centro de Granada, Instituto Aragonés de Ciencias de la Salud (IACS), Comité Etico Investigación Clínica Regional del Principado de Asturias, Comité Etico Investigación Clínica Hospital 12 de Octubre, Comité Etico Investigación Clínica Hospital Universitario Virgen de la Arrixaca, Sección de Ordenación e Inspección Farmacéutica Departamento de Salud, Comité Ético de Investigación Clínica del Hospital Universitario del Río Hortega de Valladolid, Comissão de Ética para a Saúde (CES), Centro Hospitalar de Lisboa Ocidental, EPE, Comissão de Ética para a Saúde (CES), Centro Hospitalar do Porto, EPE, Comissão de Ética para a Saúde (CES), Centro Hospitalar Lisboa Central, EPE, Comissão de Ética para a Saúde (CES), Hospital Garcia de Orta, EPE, Comissão de Ética para a Saúde (CES), Centro Hospitalar de São João, EPE, Comissão de Ética para a Saúde (CES), Hospital Professor Doutor Fernando Fonseca, EPE, Comissão de Ética para a Saúde (CES), Centro Hospitalar do Algarve, EPE (Unidade de Faro), LUHS Kaunas Regional Biomedical Research Ethics Committee, Paula Stradina kliniskās universitātes slimnicas, Attistibas biedribas Kliniskās izpētes Etikas komiteja, Ethics Committee for Clinical Research, Komisija Republike Slovenije za medicinsko etiko, Comitato Etico Indipendente Presso La Fondazione Ptv Policlinico Tor Vergata Di Roma, Comitato Etico Regione Calabria Sezione Centro c/o A.O.U. Mater Domini Di Catanzaro, Comitato Etico Azienda Ospedaliera Universitaria Di Cagliari, Comitato Etico Cardarelli-Santobono c/o Ao Cardarelli, Comitato Etico Per La Sperimentazione Clinica Delle Province Di Verona E Rovigo, Presso Aoui Verona, Eticka Komise Fn Brno, Eticka Komisia Dfnsp Bratislava, Eticka Komisia Pri Dfn Kosice, Eticka Komisia Bratislavskeho Samospravneho Kraja, Comisia Naţională de Bioetică a Medicamentului şi a Dispozitivelor Medicale, Comitato Etico Milano area 1 c/o ASST FBF Sacco - P.O. L. Sacco, Comité de Ética de la Investigación de Centro Hospital Universitario Virgen del Rocío, Comité Ético de Investigación Clínica Fundació Sant Joan de Déu Generalitat de Catalunya. Departament de Salut, Comité Ético de Investigación Clínica Hospital Infantil Universitario Niño Jesús, Consejería de Sanidad Dirección General de Salus Pública Junta de Castilla León, Dirección General de Asistencia Sanitaria, Consejería de Sanidad Gobierno del Principado de Asturias, Dirección General de Planificación, Ordenación Sanitaria y Farmacéutica e Investigación, Consejeria de Sanidad y Política Social Región de Murcia, Ethics Committee at Moscow Institute of Pediatrics and Pediatric Surgery, Paula Stradina kliniskās universitātes slimnicas, Attistibas biedribas Kliniskās izpētes Etikas komiteja, Ethics Committee for Clinical Research, The First Affiliated Hospital of The Fourth Military Medical University, Zhongshan Hospital Fudan University. Written informed consent to participate in this study was provided by the participants' legal guardian/next of kin.

## Author Contributions

RM contributed to designing the study, data analysis, data interpretation, and drafting, revising, final review, and approval of the manuscript. EB, MB, PC, MD, JF, MF, CH, SJ, JL, AM, RN, VS, MS, RT, BZ, JK, and AJ were responsible for designing the study, patient accrual, clinical care, data interpretation, and drafting, revising, final review, and approval of the manuscript. TC, VC, Gd'A, PV, CF, FO'C, JQ, YT, and SY were responsible for designing the study, data interpretation, and drafting, revising, final review, and approval of the manuscript. LD'A was responsible for designing the study, trial management, data collection, data analysis, data interpretation, and drafting, revising, final review, and approval of the manuscript. SS was responsible for designing the study, trial statistician, data analysis, data interpretation, and drafting, revising, final review, and approval of the manuscript.

### Conflict of Interest

RM and SS were employees of Novartis, while LD'A was a Novartis employee at the time of manuscript concept approval. EB, TC, VC, PC, Gd'A, PV, JF, MF, CF, CH, SJ, RN, FO'C, JQ, MS, RT, MD, JL, AM, SY, MB, BZ, JK, and AJ received honoraria and support for the travels from Novartis. VC received personal fees for consulting, lecture fees, and travel from Actelion, Bayer, Biogen Idec, Boehringer Ingelheim, Gilead, GSK, MSD, Novartis, Pfizer, Roche, Sanofi; grants from Actelion, Boehringer Ingelheim, GSK, Pfizer, Roche; personal fees for developing educational material from Boehringer Ingelheim and Roche. PV had been on the study steering group of the EXIST-1, 2, and 3 studies sponsored by Novartis, and co-PI on two investigator-initiated studies part-funded by Novartis. RN received grant support, paid to her institution, from Eisai and lectures fees from Nutricia, Eisai, Advicenne, and GW Pharma. YT received personal fee from Novartis for lecture and for copyright of referential figures from the journals, and received grant from Japanese government for intractable epilepsy research. SJ was partly financed by the EC Seventh Framework Programme (FP7/2007-2013; EPISTOP, grant agreement no. 602391), the Polish Ministerial funds for science (years 2013–2018) for the implementation of international cofinanced project and the grant EPIMARKER of the Polish National Center for Research and Development No STRATEGMED3/306306/4/2016. JK, PC, CH, JL, and JQ received research grant from Novartis. This study was funded by Novartis Pharma AG. All authors approved the final version of the manuscript prior to submission.

## TOSCA Investigators

**Japan:** Nobuo Shinohara, Shigeo Horie, Masaya Kubota, Jun Tohyama, Katsumi Imai, Mari Kaneda, Hideo Kaneko, Yasushi Uchida, Tomoko Kirino, Shoichi Endo, Yoshikazu Inoue, Katsuhisa Uruno; **Turkey:** Ayse Serdaroglu, Zuhal Yapici, Banu Anlar, Sakir Altunbasak; **Russia:** Olga Lvova, Oleg Valeryevich Belyaev, Oleg Agranovich, Elena Vladislavovna Levitina, Yulia Vladimirovna Maksimova, Antonina Karas; **China:** Yuwu Jiang, Liping Zou, Kaifeng Xu, Yushi Zhang, Guoming Luan, Yuqin Zhang, Yi Wang, Meiling Jin, Dingwei Ye, Weiping Liao, Liemin Zhou, Jie Liu, Jianxiang Liao, Bo YAN, Yanchun Deng, Li Jiang, Zhisheng Liu, Shaoping Huang, Hua Li; **Korea:** Kijoong Kim; **Taiwan:** Pei-Lung Chen, Hsiu-Fen Lee, Jeng-Dau Tsai, Ching-Shiang Chi, Chao-Ching Huang; **Australia:** Kate Riney, Deborah Yates, Patrick Kwan; **Thailand:** Surachai Likasitwattanakul, Charcrin Nabangchang, Lunliya Thampratankul Krisnachai Chomtho, Kamornwan Katanyuwong, Somjit Sriudomkajorn; **South Africa:** Jo Wilmshurst; **Israel**: Reeval Segel, Tal Gilboa, Michal Tzadok, Aviva Fattal- Valevski; **Greece:** Panagiotis Papathanasopoulos, Antigone Syrigou Papavasiliou, Stylianos Giannakodimos, Stylianos Gatzonis, Evangelos Pavlou, Meropi Tzoufi; **Netherlands:** A.M.H. Vergeer; **Belgium:** Marc Dhooghe, Hélène Verhelst, Filip Roelens, Marie Cecile Nassogne, Pierre Defresne, Liesbeth De Waele, Patricia Leroy, Nathalie Demonceau, Benjamin Legros, Patrick Van Bogaert, Berten Ceulemans, Lina Dom; **France:** Pierre Castelnau, Anne De Saint Martin, Audrey Riquet, Mathieu Milh, Claude Cances, Jean-Michel Pedespan, Dorothee Ville, Agathe Roubertie, Stéphane Auvin, Patrick Berquin, Christian Richelme, Catherine Allaire, Sophie Gueden, Sylvie Nguyen The Tich, Bertrand Godet; **Spain**: Maria Luz Ruiz Falco Rojas, Jaume Campistol Planas, Antonio Martinez Bermejo, Patricia Smeyers Dura, Susana Roldan Aparicio, Maria Jesus Martinez Gonzalez, Javier Lopez Pison, Manuel Oscar Blanco Barca, Eduardo Lopez Laso, Olga Alonso Luengo, Francisco Javier Aguirre Rodriguez, Ignacio Malaga Dieguez, Ana Camacho Salas, Itxaso Marti Carrera, Eduardo Martinez Salcedo, Maria Eugenia Yoldi Petri, Ramon Cancho Candela; **Portugal**: Ines da Conceicao Carrilho, Jose Pedro Vieira, José Paulo da Silva Oliveira Monteiro, Miguel Jorge Santos de Oliveira Ferreira Leao, Catarina Sofia Marceano Ribeiro Luis, Carla Pires Mendonca; **Lithuania:** Milda Endziniene; **Latvia:** Jurgis Strautmanis; **Estonia:** Inga Talvik; **Italy:** Maria Paola Canevini, Antonio Gambardella, Dario Pruna, Salvatore Buono, Elena Fontana, Bernardo Dalla Bernardina; **Romania:** Carmen Burloiu, Iuliu Stefan Bacos Cosma, Mihaela Adela Vintan, Laura Popescu; **Czech Republic:** Karel Zitterbart; **Slovakia:** Jaroslava Payerova, Ladislav Bratsky, Zuzana Zilinska; **Austria:** Ursula Gruber-Sedlmayr, Matthias Baumann, Edda Haberlandt, Kevin Rostasy, Ekaterina Pataraia; **United Kingdom:** Frances Elmslie, Clare Ann Johnston, Pamela Crawford; **Denmark:** Peter Uldall; **Sweden:** Paul Uvebrant, Olof Rask; **Norway:** Marit Bjoernvold, Eylert Brodtkorb, Andreas Sloerdahl, Ragnar Solhoff, Martine Sofie Gilje Jaatun; **Poland:** Marek Mandera, Elzbieta Janina Radzikowska, Mariusz Wysocki; **Germany:** Michael Fischereder, Gerhard Kurlemann, Bernd Wilken, Adelheid Wiemer-Kruel, Klemens Budde, Klaus Marquard, Markus Knuf, Andreas Hahn, Hans Hartmann, Andreas Merkenschlager, Regina Trollmann.
