# Supplementary material for: Treatment Patterns and Use of Resources in Patients With Tuberous Sclerosis Complex: Insights From the TOSCA Registry
Source: Front Neurol. 2019 Oct 25;10:1144. doi: 10.3389/fneur.2019.01144 (PMC6823684; doi:10.3389/fneur.2019.01144)
Supplement: Supplementary file 1 [file Table_1.DOCX]

Supplementary Material

Table S1. Variables of Interest in the TOSCA Registry

| General resources | Specific resources |
| --- | --- |
| Inpatient stays | |
| Total hospitalizations in last 12 months | related to: TSC, any other reason;  Number of bed days |
| Each hospitalization in the last 12 months | Hospitalization: Reason, type, procedures, treatment, length of stay, ICU-ITU stays |
| Primary care visits in the last 12 months | |
|  | related to: TSC, any other reason |
| Secondary care visits (specialists) in the last 12 months | |
|  | related to: TSC, any other reason;  Specialist: type and number of visits |
| Accident & Emergency (A&E) visits | |
| Surgical procedures | |
|  | Procedure urgency, type and complications |
| Imaging tests/procedures | |
|  | Type: CT, MRI, ultrasound, electroencephalography, angiography |
| Other tests |  |
|  | Type: molecular, genetic, blood, urine, neurophysiological, histology, biopsy, pulmonary function test (PFT), other. |
| Everolimus medication | |
|  | Dose: initial, final, mean, median  Treatment duration |
| Other medication | |
|  | Type: hormonal contraception, NSAID, ACE-inhibitors, ARB, calcineurin inhibitors, diuretic beta-blocker, alfa 1- blocker, alfa 2 – agonist, calcium antagonist, mycophenolic acid compounds, inhaled beta-2 mimetics, mTOR inhibitors (not everolimus), other |
| Comorbidities | |
|  | Type: cardiovascular, dyslipidemia, diabetes, liver disease, acute/chronic uncontrolled infections, autoinmune disorders, malignant tumours, other |
| Items that could resource in indirect costs | |
|  | Social support/rights; Contacts with TSC associations; Education (e.g. special education needs); Patient assistance requirements (e.g. assistance at home); Employment situation for adult patients; Caregiver’s working situation for children; Family members and income for adult patients; Impact of TSC on family and self; Other |

*CT****,*** *computerised tomography****;*** *MRI****,*** *magnetic resonance imaging****;*** *PTF****,*** *pulmonary function test****;*** *ACE****,*** *angiotensin-converting-enzyme; ARB****,*** *antagonist receptor blockers****;*** *AE****,*** *adverse events****;*** *TSC****,*** *Tuberous Sclerosis Complex****;*** *ICU****,*** *intense care unit****;*** *ITU****,*** *intense treatment unit****;*** *NSAID****,*** *nonsteroidal anti-inflammatory drugs****;*** *mTOR****,*** *mammalian target of rapamycin****.***

**Table S2.** Potential Analyses on the TOSCA Registry

| Use of resources based on clinical manifestation | |
| --- | --- |
| Neurological | SEGA; Cortical tuber; SEN; Cerebral white matter radial migration lines |
| Renal | Renal angiomyolipoma; Multiple renal cysts; Renal hamartoma; Impaired renal function; Renal malignancy |
| Pulmonary | Lymphangioleiomyomatosis |
| Cardiovascular | Cardiac rhabdomyoma |
| Dermatologic | ≥ 3 hypomelanotic macules; Facial angiofibroma; Shagreen patch; Ungual or periungual fibromas; Forehead plaque; Confetti lesions |
| Ophthalmologic | Retinal hamartoma |
| Epilepsy | Epilepsy |
| Use of resources based on sex | |
| Men - women | |
| Use of resources based on country | |
| Austria, Slovakia, Belgium, Slovenia, Czech Republic, Spain, Denmark, Sweden, Estonia, Australia, France, Israel, Germany, Japan, Greece, Korea, Italy, Russia, Latvia, South Africa, Lithunia, Mainland China, Netherlands, Hong Kong, Norway, Macau, Poland, Taiwan, Portugal, Thailand, Romania, Turkey. | |
| Use of resources based on country zone | |
| Europe - outside Europe | |
| Use of resources based on age at diagnosis | |
| Paediatric - adult | |
| Use of resources based on type of mutation | |
| By mutation type: TSC1 - TSC2 - no mutation diagnosed  By variation type: Pathogenic mutation - variant of unknown significance | |
| Use of resources based on time from TSC clinical diagnosis to molecular testing | |
| Use of resources based on absence/presence of prenatal diagnosis | |
| Use of resources based on absence/presence of affected relatives/TSC inheritance | |

*TSC, Tuberous Sclerosis Complex; SEGA, Subependymal giant cell astrocytoma; SEN, Subependymal nodule.*

**Table S3.** Visits to the Specialist in the Quality of Life Research Project (N=132). Analysis of healthcare visits excluding Spain (N=11) because of data inconsistencies.

|  | Visits to the specialist  (TSC-related) | Visits to the specialist  (other reason) |
| --- | --- | --- |
| Patients with no visits | 42 (31.8%) | 69 (52.3%) |
| Patients with at least 1 visit | 69 (52.3%) | 34 (25.8%) |
| Patients with 1 visit | 18 (13.6%) | 10 (7.6%) |
| Patients with 2 visits | 22 (16.7%) | 10 (7.6%) |
| Patients with ≥3 visits | 29 (22.0%) | 14 (10.6%) |
| Patients with missing or unknown number of visits | 21 (15.9%) | 29 (22.0%) |

**Table S4.** Hospitalizations over the last year in the Quality of Life Research Project (N=143)

|  | Hospitalizations |
| --- | --- |
| Patients with no hospitalizations | 101 (70.6%) |
| Patients with at least 1 hospitalization | 41 (28.7%) |
| Patients with 1 hospitalization | 25 (17.5%) |
| Patients with 2 hospitalizations | 7 (4.9%) |
| Patients with ≥ 3 hospitalizations | 9 (6.3%) |
| Patients with missing or unknown number of hospitalizations | 1 (0.7%) |

**Table S5.** Education and Use of Social Services in the Quality of Life Research Project (N=143)

| Education (children only) | Adults | Children (N=88) |
| --- | --- | --- |
| The child is not in a mainstream school | n.a | 28 (31.8%) |
| The child is in a mainstream school | n.a | 57 (64.8%) |
| *Receives special education within school* | n.a | *37/57 (64.9%)* |
| *The school offers special programs adequate to the child´s condition* | n.a | *26/57 (45.6%)* |
| Social services & benefits | Adults (N=55) | Children (N=88) |
| Disability allowance | 21 (38.2%) | 45 (51.1%) |
| Caregiver allowance | 0 (0.0%) | 10 (11.4%) |
| Psychological counselling | 3 (5.5%) | 12 (13.6%) |
| Social services | 2 (3.6%) | 4 (4.5%) |
| Social worker | 1 (1.8%) | 7 (8.0%) |
| Help completing benefit applications | 2 (3.6%) | 5 (5.7%) |
| Receives support with daily activities | 11 (20.0%) | n.a. |
| Employment and finances | Adults (N=55) | Children carers (N=88) |
| Employed | 23 (41.8%) | 58 (65.9%) |
| Unable to work due to TSC | 14 (25.5%) | 8 (9.1%) |
| TSC has impacted on the patient´s professional career | 28 (50.9%) | 50 (56.8%) |

*n.a., not available.*
